# Predictors of Mortality in Patients Successfully Weaned from Extracorporeal Membrane Oxygenation

**DOI:** 10.1371/journal.pone.0042687

**Published:** 2012-08-01

**Authors:** Wei-Wen Chang, Feng-Chun Tsai, Tsung-Yu Tsai, Chih-Hsiang Chang, Chang-Chyi Jenq, Ming-Yang Chang, Ya-Chung Tian, Cheng-Chieh Hung, Ji-Tseng Fang, Chih-Wei Yang, Yung-Chang Chen

**Affiliations:** 1 Department of Nephrology, Chang Gung Memorial Hospital, Taipei, Taiwan; 2 Division of Cardiovascular Surgery, Chang Gung Memorial Hospital, Taipei, Taiwan; 3 Chang Gung University College of Medicine, Taipei, Taiwan; D'or Institute of Research and Education, Brazil

## Abstract

**Purpose:**

Extracorporeal membrane oxygenation (ECMO) has been utilized for critically ill patients, such as those with life-threatening respiratory failure or post-cardiotomy cardiogenic shock. This study compares the predictive value of Acute Physiology, Age, and Chronic Health Evaluation II (APACHE II), Sequential Organ Failure Assessment (SOFA), and Organ System Failure (OSF) obtained on the first day of ECMO removal, and the Acute Kidney Injury Network (AKIN) stages obtained at 48 hours post-ECMO removal (AKIN_48-hour_) in terms of hospital mortality for critically ill patients.

**Methods:**

This study reviewed the medical records of 119 critically ill patients successfully weaned from ECMO at the specialized intensive care unit of a tertiary-care university hospital between July 2006 and October 2010. Demographic, clinical, and laboratory data were collected retrospectively as survival predictors.

**Results:**

Overall mortality rate was 26%. The most common condition requiring ECMO support was cardiogenic shock. By using the areas under the receiver operating characteristic (AUROC) curve, the Sequential Organ Failure Assessment (SOFA) score displayed good discriminative power (AUROC 0.805±0.055, *p*<0.001). Furthermore, multiple logistic regression analysis indicated that daily urine output on the second day of ECMO removal (UO_24–48 hour_), mean arterial pressure (MAP), and SOFA score on the day of ECMO removal were independent predictors of hospital mortality. Finally, cumulative survival rates at 6-month follow-up differed significantly (*p*<0.001) for a SOFA score≤13 relative to those for a SOFA score>13.

**Conclusions:**

Following successful ECMO weaning, the SOFA score proved a reproducible evaluation tool with good prognostic abilities.

## Introduction

Extracorporeal membrane oxygenation (ECMO) has been utilized in critically ill patients such as those with severe, reversible myocardial dysfunction and life-threatening respiratory failure. Patients on ECMO usually experience multi-organ failure and thus have high mortality [1]. Independent prognostic factors and outcome scoring systems for predicting hospital mortality for patients on the first day of ECMO support [2] and those already on ECMO support [3] have been elucidated. However, outcome predictors for patients weaning from ECMO remain unclear.

Given promising new treatment methods bridged to a ventricular assist device or transplant and limited medical resources, investigators and physicians require reliable tools for monitoring and stratifying risk for critically ill patients in clinical practice and clinical trials. However, to date no study has clarified the relationship between patients being weaned from ECMO support and their short term prognosis. Therefore, this study investigated the prognostic factors and compared the accuracy of the Acute Physiology and Chronic Health Evaluation (APACHE) II [4], Sequential Organ Failure Assessment (SOFA) [5], Organ System Failure (OSF) number [6], and Acute Kidney Injury Network (AKIN) stages [7] for predicting hospital mortality and short-term prognosis in patients being weaned from ECMO support.

## Materials and Methods

### Patient information and data collection

The Institutional Review Board at Chang Gung Memorial Hospital approved the study and waived the need for consent. All data in our study was anonymized. Between July 2006 and October 2010, 124 patients received ECMO support and experienced ECMO weaning via a 20-bed specialized cardiovascular surgery intensive care unit (CVS ICU). Those who were weaned off ECMO support but died within 48 hours (5 patients) were defined as unsuccessfully weaned and excluded. Medical records of 119 patients who were successfully weaned from ECMO support were examined.

The following retrospective data were obtained: demographic data; primary diagnosis for ECMO implementation; whether the patient was currently being weaned off ECMO support; APACHE II, SOFA score, and OSF number on the first day of ECMO support and the day of ECMO removal, respectively; AKIN stage (AKIN_48-hour_) at 48 hours post-ECMO removal; duration of hospitalization; and outcome. The primary study outcome was hospital mortality. Follow-up at 6 months after hospital discharge was performed via chart records.

### Definitions

Successful weaning was defined as weaning from ECMO support followed by survival longer than 48 hours [8]. Illness severity was assessed using APACHE II [4]. Physiological calculations utilized the worst physiological values on the first day of ECMO support and on the day of ECMO weaning. Organ function was assessed using SOFA score and OSF number, which were defined as in the original reports [5], [6]. Acute kidney injury was defined using the AKIN classification system, which requires at least two serum creatinine (SCr) values within 48 h [7]. The classification system comprises individual criteria for SCr levels and urine output (UO). A patient can fulfill the criteria by adjusting SCr concentrations, UO, or both. The baseline SCr concentration used for AKIN classification was that at the time of weaning off ECMO. The most anomalous values of each organ system on the first day of ECMO support and on the day of ECMO removal were recorded.

### Clinical management

The ECMO device (Medtronic, Inc., Anaheim, CA) comprised a centrifugal pump and a hollow-fiber microporous membrane oxygenator with an integrated heater. All ECMO circuits had a heparin-bound Carmeda bioactive surface. A hollow fiber oxygenator (Hilite LT 700; Medos, Aachen, Germany) or a silicone oxygenator (Medtronics, Minneapolis, MN, USA) was incorporated into the ECMO circuit. Percutaneous cannulation and cut-down procedures were necessary for some obese patients. According to the body size of the patient, a 17–19 Fr percutaneous arterial (outflow) cannulae and 19–21 percutaneous venous (inflow) cannulae (DLP; Medtronic Inc., Minneapolis, MN) were used. An 8 Fr distal perfusion catheter was implanted into the ipsilateral superficial femoral artery if the cannulated limb showed cyanosis.

For all patients, receiving ECMO support at least 48 hours before weaning was attempted. If the patient's hemodynamic and general condition became stable with echocardiographic evidence of improvement in left ventricular contractility (>40% of left ventricular ejection fraction), an inotropic agent was then carefully tapered. Moreover, the oxygen saturation was continuously monitored until the mixed venous oxygen saturation was more than 70% without any deterioration in hemodynamic status, and the pump flow was reduced gradually to 500 ml/min for adult patients and 20–25 ml/kg/min for pediatric patients. Finally, ECMO was withdrawn when sustained stability was noted in a patient's hemodynamic status [9].

### Statistical analysis

Descriptive statistics are expressed as means ± standard deviation (SD) unless otherwise stated. Primary analysis compared hospital survivors with non-survivors. All variables were tested for normal distributions using the Kolmogorov-Smirnov test. The Student *t*-test was applied to compare means of continuous variables and normally distributed data; otherwise, the Mann-Whitney *U* test was employed. Categorical data were tested using the χ^2^ test. Risk factors were assessed via univariate analysis, and statistically significant (*p*<0.05) variables were subjected to multivariate analysis via multiple logistic regression with forward data elimination.

Calibration was assessed using the Hosmer-Lemeshow goodness-of-fit test to compare the number of observed and predicted deaths in risk groups for the entire range of death probabilities. Discrimination was assessed using the area under the receiver operating characteristic curve (AUROC). The AUROCs were compared using a nonparametric approach. AUROC analysis was also utilized to calculate cutoff values, sensitivity, specificity, and overall correctness. Finally, cutoff points were calculated by obtaining the best Youden index (sensitivity+specificity −1). Cumulative survival curves as a function of time were generated utilizing the Kaplan-Meier approach and compared using the log rank test. All statistical tests were two-tailed, with the level of significance set to *p* less than 0.05. Data were analyzed using SPSS 13.0 for Windows (SPSS, Inc., Chicago, IL, USA).

## Results

### Subject characteristics

The study population comprised 119 patients who were successfully weaned from ECMO support at the CVS ICU between July 2006 and October 2010. Patient median age was 50 years old; 82 patients were male (69%) and 37 were female (31%). In total, 112 patients were adults (94%) and seven were children or neonates (6%). Overall in-hospital mortality was 26% (31/119). Table 1 lists patient demographic data and the clinical characteristics of both survivors and non-survivors. Table 2 lists primary diagnosis for intensive care unit admission and the primary reason for ECMO support. The most frequent indication for ECMO support in this patient subset was cardiogenic shock (77%).

**Table 1:** Patients' demographic data and clinical characteristics according to in-hospital mortality.

	All Patients (n = 119)	Survivors (n = 88)	Non-survivors (n = 31)	*p*
Age (years)	50±19	48±19	54±20	NS (0.121)
Gender (M/F)	82/37	61/27	21/10	NS (0.870)
Adult/Child	112/7	83/5	29/2	NS (1.000)
Body weight (kg)	65±19	66±19	62±19	NS (0.379)
Duration of ECMO support (h) [median]	187 [121]	169 [113]	237 [161]	0.014
Combination with CRRT on ECMO (Yes/No)	10/109	4/84	6/25	0.011
Combination with CRRT during ECMO (Yes/No)	36/83	18/70	18/13	<0.001
Combination with CRRT off ECMO (Yes/No)	27/92	12/76	15/16	<0.001
GCS, the day of ECMO removal (points)	12±4	13±3	11±4	0.003
MAP, the day of ECMO removal (mmHg)	65±12	67±12	60±10	0.003
UO, the day of ECMO removal (ml/day) [median]	2622 [2865]	2812 [2987]	2088 [1487]	NS (0.083)
UO, at post-ECMO removal 24 h (ml/day) [median]	2247 [2280]	2537 [2457]	1408 [986]	0.001
UO, at post-ECMO removal 48 h (ml/day) [median]	2167 [2320]	2485 [2610]	1270 [952]	<0.001
SCr, baseline (mg/dL) [median]	1.2 [1.1]	1.2 [1.1]	1.4 [1.0]	NS (0.861)
SCr, the day of ECMO removal (mg/dL) [median]	1.8 [1.3]	1.7 [1.2]	2.0 [1.5]	NS (0.058)
SCr, at post-ECMO removal 24 h (mg/dL) [median]	1.8 [1.3]	1.7 [1.2]	2.1 [1.6]	0.014
SCr, at post-ECMO removal 48 h (mg/dL) [median]	1.8 [1.3]	1.6 [1.1]	2.3 [1.6]	0.008
Sodium, the day of ECMO removal (mEq/L)	141±6	141±6	140±6	NS (0.467)
Albumin, the day of ECMO removal (g/L)	2.9±0.4	2.9±0.4	2.7±0.4	0.013
TnI, the day of ECMO removal (ng/mL) [median]	15 [5]	16 [6]	12 [2]	NS (0.232)
PaO_2_/FiO_2_, the day of ECMO removal (ratio)	242±142	252±135	215±161	NS (0.210)
AaDO_2_, the day of ECMO removal (mmHg)	286±150	270±148	332±150	0.050
WBC, the day of ECMO removal (×10^3^/mL)	12.9±5.4	13.1±5.4	12.4±5.4	NS (0.513)
Hb, the day of ECMO removal (g/dL)	9.9±1.0	9.9±1.0	9.7±1.2	NS (0.258)
Platelets, the day of ECMO removal (×10^9^/L)	81±33	83±32	76±36	NS (0.317)
AKIN_48-hour_ (stage 0/1/2/3) post ECMO removal*	62/18/3/30	58/11/1/16	4/7/2/14	<0.001
APACHE II score, on ECMO	23±8	22±7	25±9	0.025
APACHE II score, the day of ECMO removal	17±7	15±6	22±7	<0.001
SOFA score, on ECMO	11±3	11±3	11±4	NS (0.555)
SOFA score, ECMO removal	11±4	10±3	14±3	<0.001
OSF number, on ECMO	3±1	3±1	3±1	NS (0.569)
OSF number, ECMO removal	3±1	3±1	4±1	<0.001

Abbreviation: AaDO_2_, alveolar-arterial oxygen tension difference; AKIN, acute kidney injury network; APACHE II, acute physiology and chronic health evaluation II; CRRT: continuous renal replacement therapies; ECMO: extracorporeal membrane oxygenation; F, female; FiO_2_, fraction of inspired oxygen; GCS, Glasgow coma scale; Hb, hemoglobin; M, male; MAP, mean arterial pressure; NS, not significant; OSF, organ system failure; PaO2, partial pressure of oxygen; SCr, serum creatinine; SOFA, sequential organ failure assessment; TnI, troponin-I; UO, urine output; WBC, white blood cell count.

* , exclude 6 patients under maintenance hemodialysis.

**Table 2:** Primary diagnosis for intensive care unit (ICU) admission and extracorporeal membrane oxygenation (ECMO) support.

	All Patients	Survivors	Non-survivors	
	n (%)	n (%)#	n (%)#	*p*
*Primary diagnosis for ICU admission*				
Postcardiotomy cardiogenic shock	63 (53)	44 (50)	19 (61)	NS (0.279)
Myocarditis	9 (8)	8 (9)	1 (3)	NS (0.288)
Acute myocardial infarction	12 (10)	7 (8)	5 (16)	NS (0.194)
Decompensated heart failure	8 (7)	8 (9)	0 (0)	NS (0.082)
ARDS	23 (19)	18 (20)	5 (16)	NS (0.600)
Hypoxemia and shock	4 (3)	3 (3)	1 (3)	NS (1.000)
*Primary reason for ECMO support*				
Cardiogenic shock	92 (77)	67 (76)	25 (81)	NS (0.606)
ARDS	23 (19)	18 (20)	5 (16)	NS (0.600)
ARDS with unstable hemodynamics	4 (3)	3 (3)	1 (3)	NS (1.000)

ARDS, acute respiratory distress syndrome; NS, not significant;

# Number (%) of patients with the condition who survived or died.

### Calibration, discrimination, and correlation for illness scoring systems

Table 3 lists goodness-of-fit measured using the Hosmer-Lemeshow chi-square statistic of predicted mortality risk, and the predictive accuracy of the AKIN_48-hour_, APACHE II, SOFA score, and OSF number. Poor calibration of AKIN_48-hour_ (Hosmer-Lemeshow chi-square = 6.937, 2 df, *p* = 0.031) is noted. Table 3 also compares the discriminative abilities of these scoring systems. AUROC analysis confirms that the discriminative power of SOFA score exceeds that of the AKIN_48-hour_, APACHE II, and OSF number.

**Table 3:** Comparison of calibration and discrimination of the scoring methods in predicting hospital mortality.

	Calibration			Discrimination		
	Hosmer-Lemeshow χ^2^	df	*p*	AUROC ± SE	95% CI	*p*
MAP, off ECMO	7.004	8	0.536	0.696±0.054	0.591–0.801	0.003
SOFA score, off ECMO	5.050	7	0.654	0.805±0.055	0.698–0.911	<0.001
UO_24–48 hour_, off ECMO	10.844	8	0.211	0.761±0.057	0.650–0.872	<0.001
APACHE II, on ECMO	6.031	8	0.644	0.607±0.064	0.482–0.732	0.101
APACHE II, off ECMO	3.180	8	0.923	0.781±0.051	0.681–0.881	<0.001
OSF number, off ECMO	4.555	3	0.207	0.714±0.055	0.607–0.822	0.001
AKIN_48-hour_, off ECMO	6.937	2	0.031	0.769±0.052	0.667–0.870	<0.001

Abbreviation: AKIN, acute kidney injury network; APACHE II, acute physiology and chronic health evaluation II; AUROC, area under the receiver operating characteristic curve; CI, confidence interval; df, degree of freedom; ECMO: extracorporeal membrane oxygenation; MAP, mean arterial pressure; OSF, organ system failure; SOFA, sequential organ failure assessment; SE, standard error; UO, urine output.

### Hospital mortality and short-term prognosis

Univariate analysis identified 11 (Table 4) of 32 variables (Table 1) as prognostically valuable. Multivariate analysis identified the following variables as having independent prognostic significance: daily urine output on the second day following ECMO removal (UO_24–48 hour_), MAP, and SOFA score on the day of ECMO removal (Table 4). Regression coefficients of these variables were used to calculate a logit of death for each patient, as follows:


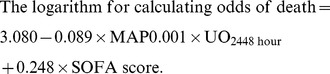


To assess the predictive value of each measure for hospital mortality, sensitivity, specificity, and overall correctness of prediction were determined. The SOFA score and AKIN_48-hour_ had the best Youden index and highest overall prediction correctness (Table 5).

**Table 4:** Variables showing prognostic significance.

Parameter	Beta Coefficient	Standard error	Odds ratios (95%CI)	*p*
Univariate logistic regression				
GCS, off ECMO first day	−0.190	0.058	0.827 (0.738–0.927)	0.001
MAP, off ECMO first day	−0.063	0.022	0.939 (0.900–0.980)	0.004
UO_0–24 hour_, off ECMO	−0.001	0.000	0.999 (0.999–1.000)	0.002
UO_24–48 hour_, off ECMO	−0.001	0.000	0.999 (0.999–1.000)	<0.001
Albumin, off ECMO first day	−1.447	0.605	0.235 (0.072–0.770)	0.017
AaDO_2_, off ECMO first day	0.003	0.001	1.003 (1.000–1.006)	0.053
AKIN_48-hour_, off ECMO	0.744	0.180	2.105 (1.479–2.996)	<0.001
APACHE II score, on ECMO	0.060	0.027	1.061 (1.006–1.119)	0.029
APACHE II score, off ECMO	0.159	0.037	1.172 (1.089–1.262)	<0.001
SOFA score, off ECMO	0.365	0.083	1.440 (1.224–1.695)	<0.001
OSF number, off ECMO	0.721	0.205	2.057 (1.376–3.075)	<0.001
Multivariate logistic regression				
MAP, off ECMO first day	−0.089	0.039	0.915 (0.848–0.988)	0.023
SOFA score, off ECMO	0.248	0.110	1.281 (1.033–1.589)	0.024
UO_24–48 hour_, off ECMO	−0.001	0.000	0.999 (0.999–1.000)	0.019
Constant	3.080	2.973	21.760	0.300

Abbreviation: AaDO_2_, alveolar-arterial oxygen tension difference; AKIN, acute kidney injury network; APACHE II, acute physiology and chronic health evaluation II; ECMO: extracorporeal membrane oxygenation; GCS, Glasgow coma scale; MAP, mean arterial pressure; OSF, organ system failure; SOFA, sequential organ failure assessment; UO, urine output.

**Table 5:** Prediction of subsequent hospital mortality after ECMO removal.

Predictive Factors	Cutoff Point	Youden Index	Sensitivity (%)	Specificity (%)	Overall Correctness (%)
MAP, off ECMO	69*	0.32	10	58	34
SOFA score, off ECMO	13*	0.51	61	90	76
UO_24–48 hour_, off ECMO	1468*	0.50	33	17	25
APACHE II, on ECMO	20*	0.30	77	52	65
APACHE II, off ECMO	18*	0.44	68	76	72
OSF number, off ECMO	2*	0.36	90	45	68
AKIN_48-hour_, off ECMO	0*	0.53	85	67	76

Abbreviation: AKIN, acute kidney injury network; APACHE II, acute physiology and chronic health evaluation II; ECMO: extracorporeal membrane oxygenation; MAP, mean arterial pressure; OSF, organ system failure; SOFA, sequential organ failure assessment; UO, urine output.

* Value giving the best Youden index.

**Figure 1:**
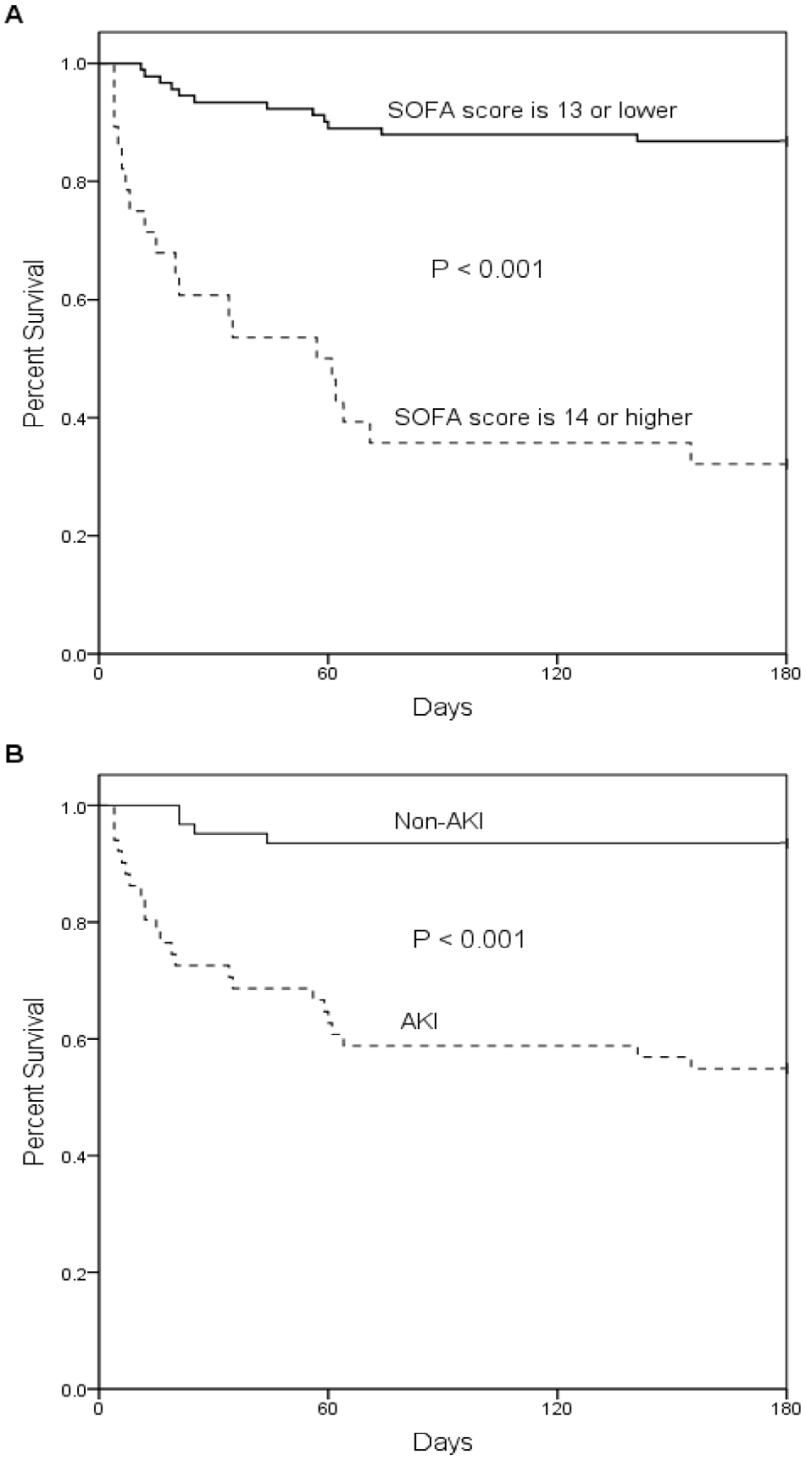
Cumulative survival rate for 119 critically ill patients based on (A) sequential organ failure assessment (SOFA) score on the day of ECMO removal and (B) acute kidney injury (AKI) or not based on changes over the course of 48 hours after ECMO removal. AKI defined as in the AKIN_48-hour_ classification system with stage 1, 2 and 3.

Figure 1A shows the cumulative rates of survival for the study group dichotomized by 13 SOFA points or less/14 SOFA points or more (*p*<0.001). Cumulative survival rates differed considerably (*p*<0.001) between non-AKI and AKI (Figure 1B).

## Discussion

The hospital mortality rate for patients who received ECMO support around 60% [1]–[3] and experienced successful ECMO weaning in this study was 26%. This investigation demonstrates that MAP and SOFA score on the day of ECMO removal, and daily urine amount on the second day after weaning from ECMO, were strong predictors of in-hospital mortality (Table 4). The SOFA score had better discriminatory power than the AKIN_48-hour_, APACHE II and OSF number (Table 3). Moreover, the SOFA score and AKIN_48-hour_ had the best Youden index and highest overall prediction accuracy (Table 5).

Urine volume is a more sensitive marker for early detection of AKI than serum creatinine level. Decreased urine volume on the day of ECMO removal is attributed to decreased cardiac output following decannulation, and is correlated with acute cardiorenal syndrome (CRS type 1) [10]. For patients with improved systolic function, urine volume may increase gradually in the following days; for other patients, decreased urine volume progresses and causes fluid overload, which likely increases preload and may contribute to circulatory failure. Additionally, loop diuretics were usually prescribed for better diuresis in cases of decreased urine volume. Metra *et al.* [11] identified the use of loop diuretics, probably by further activation of the renin-angiotensin-aldosterone system and possibly worsening intra-renal hemodynamics, as one of the modifiable in-hospital determinants of CRS type 1. Circulatory failure may further aggravate in-hospital mortality. For patients weaned from ECMO, this study adopted the best Youden index, and established a cut-off (urine volume of 1468 ml) (Table 5). Hospital mortality rates below and above the cutoff value of 1468 ml of daily urine volume on the second day of ECMO removal were 59.5% (22/37) and 12.5% (9/72) (*p*<0.001), respectively.

Mehta and colleagues [12] reported that blood pressure is a key bedside tool for predicting postoperative dialysis risk in patients undergoing cardiac surgery. Hypotension is related to worsening renal function for patients weaned from ECMO support. Damaged cardiac function leads to low cardiac output and, therefore, hypoperfusion, and may precipitate pre-renal AKI, which, if not promptly corrected, can evolve into intrinsic AKI and even cortical necrosis, resulting in irreversible loss of renal function [13], [14]. Analytical results demonstrate that MAP on the day of ECMO removal is an independent risk factor for patients weaned from ECMO. This investigation applied the best Youden index and a recognized MAP cut-off value of 69 mmHg (Table 5). Hospital mortality rates differed considerably according to the best Youden index was below or above the cutoff of 69 mmHg of MAP (34.6% *vs.* 9.8%, *p* = 0.003).

Ceriani *et al.* [15] concluded that SOFA was applicable in cardiac surgery without requiring specific modifications based on the good results they obtained in 218 patients. Belohlavek and colleagues [16] reported that patients who died on ECMO had higher SOFA scores (14.8±1.6 vs. 10.8±1.5; *p* = 0.0065). Wu *et al.* [17] also stated that SOFA score before ECMO implantation exceeding 14 predicted mortality. For patients successfully weaned from ECMO, this study adopted the best Youden index, and established a cut-off value of 13 for SOFA score (Table 5). Hospital mortality rates differed significantly below and above this cut-off value (13.2% *vs.* 67.9%, *p*<0.001). This study demonstrated that SOFA score exceeding 13 on the day of ECMO removal predicted hospital mortality, a result compatible with previous studies.

Acute kidney injury following cardiac surgery is a well-recognized complication that occurs in up to 40% of patients and requires dialysis in 1% of cases [18]. Patients who develop AKI have high mortality and resource utilization. Emerging evidence suggests that even small increases in creatinine following cardiac surgery are associated with significantly increased mortality. Following weaning from ECMO for 48 hours, hospital mortality rates differed significantly between patients with AKI *vs.* those without AKI (45.1% *vs.* 6.5%, *p*<0.001).

Despite the promising study results, this study has several important limitations. First, this was a retrospective study performed at a single tertiary-care medical center, limiting the generalizability of the findings. Second, this retrospective study suffered difficulties associated with the unavailability of certain laboratory data, including serum sodium, albumin, and troponin-I levels. Third, the patient group comprised patients weaned from ECMO excluding those who died within 48 hours of weaning; therefore, our results may not be directly extrapolated to other patient populations. Furthermore, the prognostic markers results are only applied for patients surviving 48 hours of ECMO removal. Fourth, the patient population contained a high proportion of cardiogenic shock patients (77%) (Table 2) and may present as a special subgroup of patients on ECMO. Besides, different prognostic markers might be identified in ARDS patients (23%) weaned from ECMO support, such as alveolar-arterial O_2_-tension difference or PaO2/FiO2 ratio. Fifth, because of the relatively small sample size, sensitivity, specificity and the predictive accuracies derived from the “best cut-off point” require further external validation. Sixth, the predictive accuracy of logistic regression models has its own limitations. Finally, sequential measurement of these scoring systems (*such as*, daily or weekly) may reflect the dynamic aspects of clinical diseases, thus providing superior information for outcome prediction.

In conclusion, this study observed a hospital mortality rate of 26% in critical ill patients weaned from ECMO support. According to the analytical results, the risk of mortality increases with decreasing urine volume, low MAP, and high SOFA score at the time of ECMO removal. Our data also demonstrate the good discriminative power of SOFA score to predict hospital mortality of critically ill patients weaned from ECMO support. We recommend physicians use SOFA score to assess short-term prognosis in this subset of patients.
